# Commentary on “In-Stent Restenosis in Peripheral Arterial Disease: Ultra-High-Resolution Photon-Counting Versus Third-Generation Dual-Source Energy-Integrating Detector CT Phantom Study in Seven Different Stent Types” Dachs TM, et al. CVIR 2024

**DOI:** 10.1007/s00270-024-03929-0

**Published:** 2024-12-10

**Authors:** Josua A. Decker, Thomas Kroencke

**Affiliations:** Department of Diagnostic and Interventional Radiology, University Hospital Augsburg, Stenglinstr. 2, 86156 Augsburg, Germany

## Commentary

Photon-counting detector (PCD) CT offers multiple advancements over traditional energy-integrating detector (EID) CT, including improved spatial resolution, increased contrast-to-noise ratio, and inherent spectral capabilities. These features have shown promise in enhancing diagnostic accuracy across various clinical applications [1]. In their recent phantom study, Dachs et al. make a valuable contribution to this field by applying ultra-high-resolution PCD-CT to visualize in-stent restenosis (ISR) in peripheral arterial disease (PAD) in a direct comparison with third-generation dual-source EID-CT [2].

A key finding of the study is that while PCD-CT performed comparably to EID-CT for stenoses up to 75%, its superiority became evident in high-grade ISR. This advantage likely stems from the narrow lumen in severe stenosis, where PCD-CT’s higher spatial resolution enhances lumen delineation. These results align with prior studies which showed that PCD-CT provides the most significant imaging benefits for very small stents (2.5–4 mm), which are more commonly used in coronary arteries [3, 4]. Although lower extremity stents are generally larger, the findings suggest that the narrow luminal conditions of high-grade ISR may benefit similarly from PCD-CT’s precision when applying its 0.2-mm ultra-high-resolution (UHR) acquisition mode.

The study provides an outlook on how reliable imaging of high-grade ISR with UHR PCD-CT could play a critical role in reducing unnecessary diagnostic procedures and facilitating faster, more targeted treatment decisions. By providing clearer visualization of challenging cases, PCD-CT may help streamline pathways of patients with PAD, minimizing delays and avoiding additional diagnostic steps that may carry risks and potential complications. This aligns with findings from current research in stable chest pain patients, where PCD-CTs improved and more accurate visualization of small stenoses demonstrated the potential to reduce unnecessary additional diagnostic procedures [5]. As noted in this cost-effectiveness study, these improvements not only benefit patient care but may also lead to overall reductions in healthcare expenditures by optimizing resource use in high-demand clinical settings.

While the results are promising, the reliance on a phantom model is an inherent limitation of this study. Phantom studies offer a controlled environment for evaluating imaging performance but do not replicate the complexities of in vivo conditions. Future patient-based studies are essential to validate these findings and determine to which extent the observed advantages influence clinical outcomes.

In conclusion, the work of Dachs et al. highlights the potential of PCD-CT to address specific challenges in ISR imaging in PAD, particularly in cases of high-grade stenosis. While the study demonstrates clear advantages of UHR imaging, its findings must be validated in clinical settings to account for varying physiological and anatomical conditions. Additionally, understanding the precise clinical scenarios where UHR PCD-CT offers the greatest value—such as reducing unnecessary invasive procedures or streamlining treatment pathways—will be key to its broader adoption. As research advances, PCD-CT may become a critical tool in tailored patient management, complementing existing diagnostic approaches and potentially refining care strategies for PAD patients.

## References

[1] Douek PC, Boccalini S, Oei EH, et al. Clinical applications of photon-counting CT: a review of pioneer studies and a glimpse into the future. Radiology. 2023. 10.1148/radiol.222432. 37787672 10.1148/radiol.222432 PMC10623209

[2] Dachs TM, Hauck SR, Kern M, et al. In-stent restenosis in peripheral arterial disease: ultra-high-resolution photon-counting versus third-generation dual-source energy-integrating detector CT phantom study in seven different stent types. Cardiovasc Intervent Radiol. 2024. 10.1007/s00270-024-03874-y. 39500749 10.1007/s00270-024-03874-y PMC11706893

[3] Decker JA, O’Doherty J, Schoepf UJ, et al. Stent imaging on a clinical dual-source photon-counting detector CT system: impact of luminal attenuation and sharp kernels on lumen visibility. Eur Radiol. 2023;33(4):2469–77. 10.1007/s00330-022-09283-4. 36462045 10.1007/s00330-022-09283-4

[4] Hagar MT, Soschynski M, Saffar R, et al. Ultra-high-resolution photon-counting detector CT in evaluating coronary stent patency: a comparison to invasive coronary angiography. Eur Radiol. 2024;2024:1–11. 10.1007/s00330-023-10516-3. 10.1007/s00330-023-10516-3 PMC11213791 38177617

[5] Vecsey-Nagy M, Emrich T, Tremamunno G, et al. Cost-effectiveness of ultrahigh-resolution photon-counting detector coronary CT angiography for the evaluation of stable chest pain. J Cardiovasc Comput Tomogr. 2024. 10.1016/j.jcct.2024.10.011. 39500702 10.1016/j.jcct.2024.10.011

